# Early identification of DPAM in at-risk low-grade appendiceal mucinous neoplasm patients: a new approach to surveillance for peritoneal metastasis

**DOI:** 10.1186/s12957-016-0996-0

**Published:** 2016-09-13

**Authors:** Jason M. Foster, Richard L. Sleightholm, Steve Wahlmeier, Brian Loggie, Poonam Sharma, Asish Patel

**Affiliations:** 1 Division of Surgical Oncology, Department of Surgery, University of Nebraska Medical Center, 984030 Nebraska Medical Center, Omaha, NE 68198-4030 USA; 2 Division of Surgical Oncology, Department of Surgery, Alegent Creighton University Health Center, Omaha, USA; 3 Department of Pathology, Alegent Creighton University Health Center, Omaha, USA

**Keywords:** Appendiceal, Pseudomyxoma peritonei, Perforated appendicitis

## Abstract

**Background:**

Disseminated peritoneal adenomucinosis (DPAM) patients often have a history of appendectomy with identification of an incidental mucinous neoplasm (low-grade appendiceal mucinous neoplasm (LAMN)). The rate of developing DPAM is not well established.

**Methods:**

Twenty-two patients with incidental LAMN were identified and monitored with cancer markers and CT every 4–6 months. Laparoscopy with peritoneal washing was performed in patients either in the event of radiographic disease or after 12 months in absence of radiographic disease. The rate of detecting peritoneal metastasis was determined for CT scan and laparoscopy.

**Results:**

Peritoneal metastasis was detected in 5 (23 %) patients. Occult disease was detected in four patients at laparoscopy without a detectable disease on CT scan. One patient developed radiographic progression at 6 months confirmed with laparoscopy. Four patients were treated with cytoreductive surgery (CRS)/HIPEC and one with CRS only. The 17 patients with negative laparoscopy remain disease free with a median follow-up of 50 months.

**Conclusions:**

The rate of peritoneal metastasis in incidental LAMN patients was 23 %. Laparoscopy was the primary screening tool identifying occult metastasis. The median PCI of 7 was low, and all the patients underwent R0/R1 resections. This study revealed 1 in every 4.4 patients with LAMN may develop PMP. Longer follow-up and further patient surveillance is warranted.

## Background

Appendix tumors are rare, representing less than 1 % of gastrointestinal cancers and incidentally detected in approximately 1 % of appendectomy specimens [1–4]. The two most common histologies identified are mucinous and carcinoid tumors [5, 6]. Carcinoid tumors, like colorectal cancer, have a propensity for lymphatic and hematogenous spread [7]. Conversely, mucinous tumors infrequently exhibit hematogenous spread, and nodal involvement is rare in low-grade mucinous tumors [8, 9]. Peritoneal dissemination is the most common form of metastasis observed in low-grade appendiceal mucinous neoplasms (LAMN), and this condition is called pseudomyxoma peritonei (PMP). PMP has been split into low- and high-grade diseases which has been further subclassified by Ronnett into disseminated peritoneal adenomucinosis (DPAM) (low grade), peritoneal mucinous carcinomatosis (PMCA)-I (intermediate grade), and PMCA (high grade) which characterize PMP along a continuum of biological behavior [9].

Majority of patients who present with low-grade PMP are symptomatic with bulky peritoneal disease [10]. The slow-growing non-invasive biology of these tumors contributes to the development of extensive peritoneal disease before patients seek medical attention for clinical symptoms. This indolent pattern of growth not only affords patients with PMP an opportunity for symptom palliation but majority can achieve long-term remission with cytoreductive surgery and hyperthermic intraperitoneal chemotherapy (CRS/HIPEC) [11].

Cytoreductive procedures often require extensive operative time and extended recovery. The factors predicting long-term survival with DPAM are well established and include disease extent measured by peritoneal cancer index (PCI), ability to achieve optimal cytoreduction quantified by completeness of cytoreduction (CC) score or R score (CC-0/CC-1 or R0/R2a), and early referral to centers with experience in CRS surgery [12–14].

Optimal tumor cytoreduction can be achieved in 70–90 % of DPAM patients, but this typically requires extensive cytoreductive procedures with multivisceral organ resections, prolonged hospital stay, and post-operative morbidity and mortality rates of 30–50 % and 3–7 %, respectively [13]. Many patients who develop symptomatic low-grade PMP have a history of appendectomy with an incidental LAMN identified. Given the low incidence of LAMN tumors, there is a paucity of data that defines the rate of LAMN progression to PMP of the DPAM subtype, and like most rare tumors, there is currently no consensus on follow-up guidelines. In spite of these obstacles, the development of a surveillance strategy in LAMN may not only provide insight into the rate of PMP development but facilitate early detection of occult peritoneal metastasis in LAMN patients and improve outcomes in PMP patients. Currently, only one retrospective population study in the Netherlands reported an observed PMP incidence of 20 % in 547 patients with LAMN lesions. Majority of the PMP events were detected within 24 months, but in a few cases, PMP occurred after 2 years [15]. As a result, this group recommends performing cross-sectional imaging for at least 5 years in patients with incidental LAMN tumors. Since CT imaging has known limitations in the detection of peritoneal metastasis, a second modality, diagnostic laparoscopy, was added to the surveillance regimen to determine if occult disease could be identified in patients in the absence of CT-detectable disease. Here, we report the results of this surveillance strategy.

## Methods

### Patient population

A retrospective chart review was conducted on patients referred for management of LAMN. In this study, low-grade mucinous tumors were defined by the grading system reported by Carr et al. and Misrdraji et al., recently adopted by the Peritoneal Surface Oncology Group International (PSOGI) [16–19]. All cases included in this analysis had mucin with low-grade architectural features, “pushing alteration” of the muscularis mucosa or wall, and when pathology was reviewed at our institutions, the presence of perforation, extra-appendiceal mucin, and extra-appendiceal cells was reported. Twenty-two LAMN patients were identified, diagnosed between June 2006 and May 2013, and comprehensive pathology reports were available in 20 out of the 22 patients. In two LAMN cases, the comprehensive pathology reports were not available.

### Surveillance strategy

**Fig. 1:**
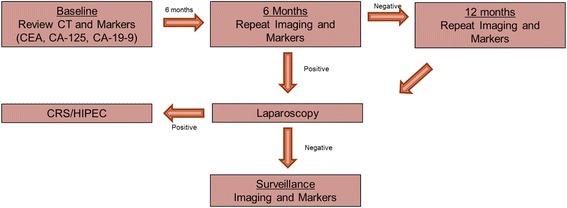
Surveillance algorithm for LAMN patients

All patients had a baseline CT scan performed prior to their appendectomy, and these images were reviewed at first consultation (Fig. 1). All outside medical records and pathology and imaging reports were reviewed. Baseline tumor markers were obtained at the first consultation which included CA-19-9, CEA, and CA-125. The patients were re-evaluated every 4–6 months with clinical exams, abdomen and pelvis CT scans, and tumor markers. If any radiographic abnormality was reported by the reading radiologist consistent with concerns of peritoneal metastasis, patients were taken for diagnostic laparoscopy to pathologically document disease progression. After 12 months of surveillance, all the patients underwent diagnostic laparoscopy to determine if occult peritoneal metastasis could be identified in the absence of CT-detectable disease. At the time of laparoscopy, PCI zones were evaluated for peritoneal implantation and peritoneal washings were performed. Any suspicious nodules identified were biopsied and sent for pathological evaluation. If pathologically confirmed peritoneal disease was detected, the patient was offered definitive CRS/HIPEC. All the patients with positive findings elected to undergo CRS/HIPEC except for one patient who only underwent CRS without HIPEC.

### Clinical and outcomes data analysis

Demographic information was collected on all LAMN patients including age, sex, and additional procedures performed prior to referral and any administration of chemotherapy by referring providers. The appendectomy proximal margin was negative in all 22 specimens, and in 20/22 cases, data was available to determine the presence of perforation or extra-appendiceal mucin and/or extra-appendiceal cells. Following diagnostic laparoscopy, adverse events, cytology results, and rate of detection of peritoneal implants were determined. Finally, we calculated median follow-up time, overall survival, and current status of disease.

In LAMN patients where peritoneal metastasis was identified, all underwent CRS/HIPEC except for one patient who underwent CRS alone. PCI score (tumor burden) [20], CC score, length of stay (LOS), overall survival, and disease-free survival status were determined. Right hemicolectomy, partial peritonectomy sites and rates, visceral organ resection rates, and total resections performed which included peritonectomy procedures were also calculated. The organ resections performed included omentectomy, right colectomy, cholecystectomy, distal pancreatectomy, splenectomy, and non-anatomical hepatectomy or liver capsule resection. All peritonectomies were limited partial resections, and the sites included the diaphragm, colic gutters, and pelvis. Morbidity and mortality were quantified by the Clavien-Dindo classification grades I–V [21].

## Results

### Demographics of surveillance group and serology results

The mean age at diagnosis for the 22 LAMN patients was 53, and 55 % (12) were males. Twenty-one patients (95 %) underwent appendectomy for their disease. One patient (5 %) underwent right hemicolectomy and cholecystectomy and was also started on chemotherapy prior to referral. Following diagnostic laparoscopy, all the patients were discharged within 23 h of the procedure without any adverse events. In all the 22 patients, CEA, CA-19-9, and CA-125 levels were within normal limits and no patient experienced any marker elevation.

#### LAMN appendix pathology features, rates and sites of PM implants, and cytology results

**Fig. 2:**
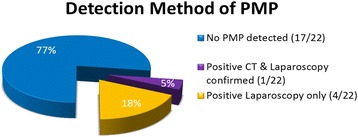
Incidence of detection of peritoneal metastasis in LAMN patients

Comprehensive pathological characteristics were available in 91 % (20/22) of the patients (Table 1). Appendix perforation and extra-appendiceal mucin were identified in 90 % (18/20) of the specimens. In these 18 patients with perforation and mucin extravasation, 5/18 (28 %) developed peritoneal metastasis while 13/18 (72 %) had no evidence of peritoneal metastasis at diagnostic laparoscopy. Extra-appendiceal cells were identified in 10/20 of the patients, 4/10 (40 %) developed peritoneal metastasis while 6/10 (60 %) had no evidence of peritoneal metastasis. The overall rate of peritoneal metastasis was 5/22 (23 %) (Fig. 2); perforation and extra-appendiceal mucin were present in 5/5 (100 %) of the patients, and extra-appendiceal cells were present in 4/5 (82 %) of the patients (Table 1). At laparoscopy, cytology was positive for cells in 60 % (3/5) of the patients with peritoneal metastasis (Table 1).

**Table 1:** Individual descriptive appendectomy pathology and cytology results at the time of laparoscopic surveillance

LAMN without DPAM	Perforation	Extra-appendiceal mucin	Extra-appendiceal cells	Cytology at laparoscopy
1	+	+	−	−
2	+	+	+	−
3	+	+	+	−
4	+	+	−	−
5	+	+	+	−
6	+	+	+	−
7	+	+	+	−
8	+	+	−	−
9	+	+	−	−
10	−	−	−	−
11	+	+	−	−
12	−	−	−	−
13	+	+	+	−
14	+	+	−	−
15	+	+	−	−
16	N/A	N/A	N/A	−
17	N/A	N/A	N/A	−
Total	13/15 (87 %)	13/15 (87 %)	6/15 (40 %)	0/17 (0 %)
LAMN with DPAM	Perforation	Extra-appendiceal mucin	Extra-appendiceal cells	Cytology at laparoscopy
1	+	+	+	+
2	+	+	−	−
3	+	+	+	−
4	+	+	+	+
5	+	+	+	+
Total	5/5 (100 %)	5/5 (100 %)	4/5 (80 %)	3/5 (60 %)
All patients	18/20 (90 %)	18/20 (90 %)	10/20 (50 %)	3/22 (14 %)

Descriptive pathology results were not available in two patients labeled N/A

**Fig. 3:**
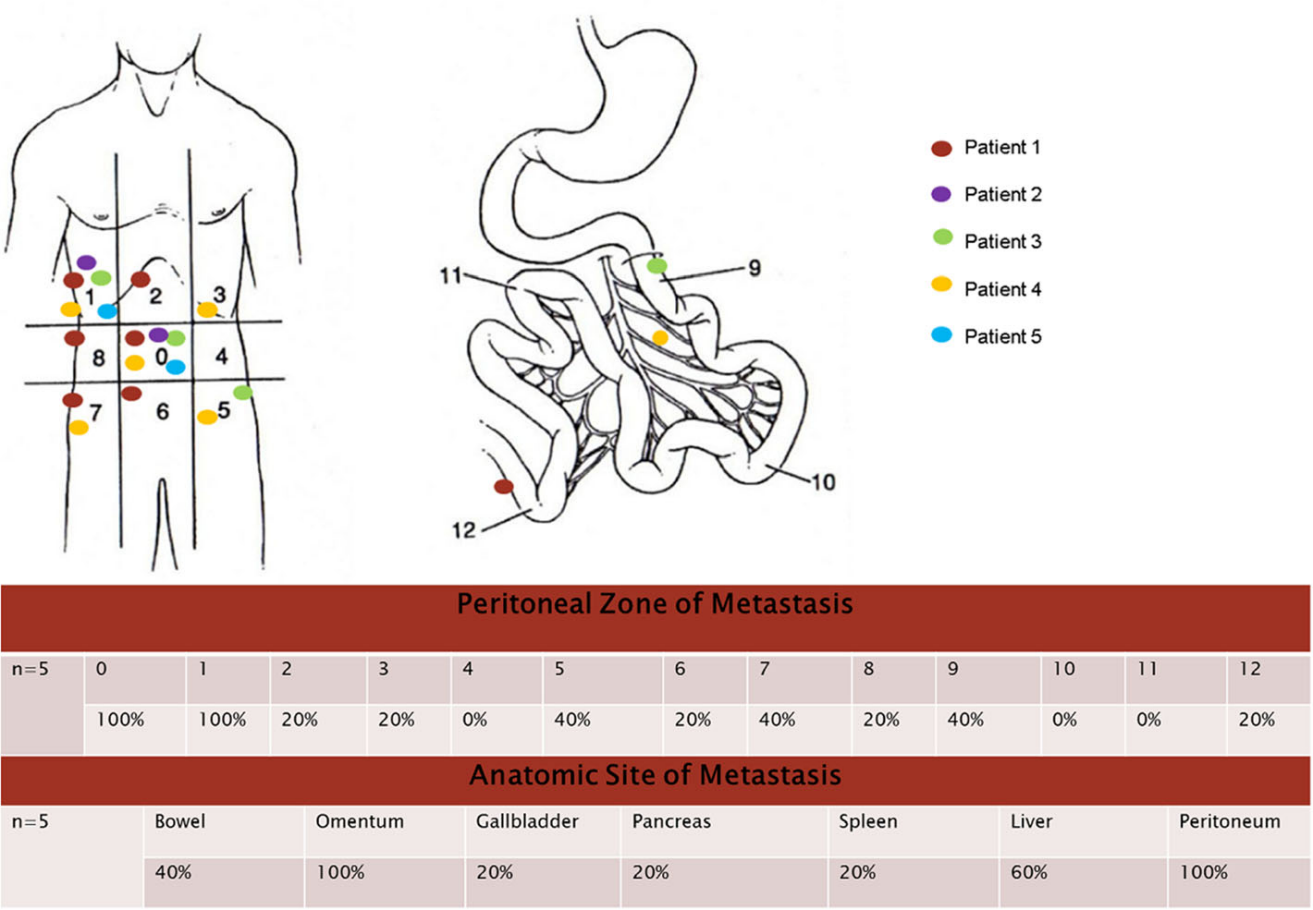
Map and table of metastasis based on PCI zones

The most frequent PCI zones with occult metastases were zones 0 and 1 with disease identified in all 5 patients (100 %). Figure 3 provides a comprehensive description of the sites involved for each patient and the rate of involvements in each PCI zone.

### Outcomes in LAMN patients without PMP

All 17 patients had no evidence of disease or pathology on CT imaging, tumor marker serology was within normal limits, and there was no detectable tumor at laparoscopy. Tumor cytology in this group of patients was negative for tumor or mucin in peritoneal washings. One patient underwent a second laparoscopy at the 2-year mark because of inflammatory changes found at the first laparoscopy, and biopsies at the first laparoscopy revealed inflammatory nodules. The second laparoscopy revealed resolution of the inflammatory changes, and no disease or inflammation was identified. The median follow-up time was 50 (20–106) months, and none of the patients developed clinical progression during the follow-up period.

### Outcomes in LAMN patients with PMP

Five patients (23 %) were identified who developed peritoneal metastasis (Fig. 2). The patient that underwent right hemicolectomy (RHC) and cholecystectomy and received FOLFOX chemotherapy prior to referral had radiographic progression and evidence of DPAM at 6 months following LAMN diagnosis with evidence of omental nodularity. Laparoscopy was performed based on the CT scan and confirmed peritoneal metastasis. The radiographic PCI in this case was 8, and the PCI at exploration was 14 (Table 2, patient #4). In the other four cases with negative CT results, occult peritoneal disease was identified at laparoscopy. The sites of detected disease can be found in Fig. 3.

**Table 2:** RHC status, PCI score, organs resected, LOS, and CC score for the LAMN group with PMP

LAMN with DPAM	RHC	PCI	# of organs resected	Peritonectomies	LOS	CC score	HIPEC	Recurrence
1	+	8	2	2	7	1	+	−
2	−	7	2	2	7	0	+	−
3	−	5	1	3	6	0	−	+
4	+^a^	14	2	3	8	1	+	−
5	−	7	1	4	8	1	+	−

All RHC specimens were negative. All peritonectomies were partial resection not complete in PCI zones

^a^Indicates RHC was done prior to CRS/HIPEC

The rate of RHC was 40 % (2/5), with one performed prior to referral and one performed at the time of CRS/HIPEC because of concerns for gross surface disease (Table 2). In both cases, the pathological analysis of the specimens revealed no surface or nodal involvement. In all cases (5/5), a CC-0/CC-1 was achieved, and Table 2 provides each patient’s PCI scores, organ resections, peritonectomy rates, and LOS, while Fig. 3 provides the PCI zones of disease detection for each patient. Median PCI and total organ resection values were 7 and 3, respectively, reflecting the limited disease burden. The median LOS was 7 days, and no post-operative deaths occurred with only one patient experiencing a grade II Clavien-Dindo adverse medical event, hypertension requiring an esmolol drip.

In 4/5 patients treated with CRS/HIPEC, all the patients are alive without disease recurrence with median follow-up of 50 months. In the patient who elected to have CRS alone, disease recurrence occurred at 24 months after cytoreduction (36 months from diagnosis). The recurrence was detected on CT imaging, and the patient had no clinical symptoms. The patient underwent a second procedure with a PCI score of 19, and CC-1 cytoreduction was achieved. The patient is currently alive without recurrence or progression.

## Discussion

The optimal management of incidental LAMN tumors had been a challenging area to define because of the low incidence and indolent behavior of LAMN tumors that develop peritoneal metastasis resulting in low-grade PMP, DPAM. The goal of this study was to longitudinally follow patients with only LAMN tumors, excluding all other histologies, and evaluate these patients for the development of peritoneal metastasis with imaging and laparoscopy. It has been established that low-grade mucinous appendix tumors are the most common primary tumor site resulting in the development of low-grade PMP, with very minimal risk of nodal or hematogenous dissemination [22]. What has not been clearly established is the rate at which LAMN tumors disseminate and develop low-grade PMP. In this series of 22 patients, occult peritoneal metastasis was detected in 5 (23 %) patients. Only one case was detected on imaging and confirmed with laparoscopy, while 80 % (4/5) of the cases were only detected by laparoscopy. Although this is a small series, this observed rate of PMP is consistent with the rate reported by Smeenk et al., where a PMP rate of 20 % was observed in patients with LAMN histology [15]. This correlation of event rates of PMP development provides support that laparoscopy may be a useful screening tool for LAMN patients given the limitations of the small sample size.

Current surveillance recommendations for the management of low-grade mucinous appendix tumors range from no routine imaging to imaging every 6–12 months, or even consideration of CRS/HIPEC for patients with perforated disease and extravasated mucin. This data revealed that a CT scan as a surveillance tool may be inadequate for the detection of early PMP. This is consistent with other data demonstrating the CT scan often under-stages the volume of peritoneal disease in patients with known peritoneal metastasis [19]. MRI was not performed in these patients, but recent data demonstrates it may be a more sensitive tool for detection of peritoneal metastasis [23, 24]. As a result, we have switched to patients and only perform CT imaging when patients cannot tolerate MRI or when motion artifacts may limit interpretation.

It has been suggested in the setting of perforated appendix with extra-appendiceal mucin and/or cells in LAMN patients consider CRS/HIPEC [25]. The comprehensive pathological analysis revealed that these features were present in all patients who develop PMP; however, this feature was present in 90 % (18/20) of all the specimens. Specifically, 13/18 patients with these features were identified without detectable occult peritoneal metastasis at laparoscopy. If CRS/HIPEC was performed for all patients with perforation and extra-appendiceal mucin, 72 % (13/18) of the patients with this feature potentially may have been over-treated and subjected to the risks of CRS/HIPEC. A similar result was observed in patients with extra-appendiceal cells where it was identified in 10/20 cases and 60 % (6/10) of the patients would have been treated with CRS/HIPEC that did not develop DPAM. Delayed recurrences are a real concern, and continued follow-up is important. However, none of the 17 patients without evidence of occult peritoneal metastasis have developed PMP with a median follow-up period of 50 months, and they continue to undergo active imaging surveillance every 12 months.

Laparoscopy appears to be a promising screening tool in LAMN patients, but it is an invasive procedure with surgical risks. In this series, no major adverse events occurred, but bowel injury, port site infections, and hernia are complications that must be balanced with the benefits of early detection. Larger patient series will be necessary to address this question. There are limitations to this study including the small sample size and retrospective data collection. In spite of these limitations, the observed rate of PMP is consistent with the rate reported in the literature based on a study consisting 574 patients with LAMN, wherein 114 (20 %) developed PMP, and this provides some validation of the results observed [15]. The benefits of early detection include asymptomatic detection resulting in low PCI index at surgery, 100 % optimal cytoreduction rate, and potentially a small surgical procedure evident by a low rate of organ resections. The impact on survival is not clear since the current follow-up is only 50 months, but given the favorable factors of low PCI and CC-0/CC-1 in all cases, this would support a more favorable outcome [14]. Interestingly, the only recurrence occurred in one patient who did not receive HIPEC after 3 years of follow-up. It is not clear what other factors played a role in this patient’s recurrence, but this observation provides evidence that these occult tumors are biologically active and can progress/recur over time. This patient is currently in remission following the second CRS with HIPEC.

Based on this preliminary data, our group is establishing a prospective registry trial employing this surveillance protocol utilizing cross-sectional imaging (MRI or CT) every 4–6 months and serological marker assessment and offering all patients laparoscopy after 1 year of imaging surveillance or in the event of radiologic findings of peritoneal metastasis. Through a multi-institutional collaborative effort, we hope that continued research in this area will more clearly define the incidence of PMP and result in early detection of the syndrome.

## Conclusions

The rate of peritoneal metastasis in incidental LAMN patients was 23 %, and laparoscopy was the primary screening tool identifying occult metastasis. Given the favorable prognosis of early detection and treatment of PMP, DPAM, further trials utilizing a surveillance strategy of laparoscopy, serological markers, and cross-sectional imaging are warranted.

## Abbreviations

CRS: Cytoreductive surgery
CC: Completeness of cytoreduction
CT: Computed tomography
DPAM: Disseminated peritoneal adenomucinosis
HIPEC: Hyperthermic intraperitoneal chemotherapy
LOS: Length of stay
LAMN: Low-grade appendiceal mucinous neoplasm
PCI: Peritoneal cancer index
PMCA: Peritoneal mucinous carcinomatosis
PMP: Pseudomyxoma peritonei
RHC: Right hemicolectomy
